# Automatic adaptive radiotherapy triggering based on CBCT using deep learning for esophageal cancer underwent volumetric modulated arc therapy

**DOI:** 10.1002/acm2.70651

**Published:** 2026-06-10

**Authors:** Juebin Jin, Jiayi Xu, Yapeng Liu, Kai Shao, Long Zhang, Yangyang Zhang, Ce Han, Huawei Yan, Zhiwei Wang, Li Lin, Congying Xie, Xiance Jin, Yongqiang Zhou

**Affiliations:** ^1^ Department of Medical Engineering 1st Affiliated Hospital of Wenzhou Medical University Wenzhou China; ^2^ Department of Radiation and Medical Oncology 1st Affiliated Hospital of Wenzhou Medical University Wenzhou China; ^3^ Department of Radiation Oncology The Affiliated Suzhou Hospital of Nanjing Medical University Suzhou Municipal Hospital Suzhou China; ^4^ School of Basic Medical Science Wenzhou Medical University Wenzhou China

**Keywords:** adaptive radiotherapy, automatic segmentation, cone beam CT, deep learning, dose prediction

## Abstract

**Background:**

Esophageal cancer (EC) is a highly fatal malignancy for which radiotherapy plays a critical role in treatment. However, anatomical changes during radiotherapy can lead to increased doses to surrounding organs at risk (OARs). Adaptive radiotherapy (ART) is a strategy that incorporates patient‐specific anatomic changes into treatment plan modification to minimize overdose to surrounding healthy tissues. Identifying appropriate triggers with in‐room verification imaging is critical to maximize the benefits of ART and prevent additional imaging dose to a certain extent for patients without compromising clinical resources or operations.

**Purpose:**

To develop an automatic ART triggering procedure by predicting the patient's anatomical changes and their resulted dose‐volume metrics differences on OARs using deep learning (DL) based on cone‐beam computed tomography (CBCT) to efficiently and effectively balance the benefit and frequency of ART.

**Methods:**

A DL network was first trained to automatically segment OARs on the CBCT of 136 EC patients underwent volumetric modulated arc therapy (VMAT). The dose distributions on CBCT with the automatically contoured OARs were predicted with Unet. A set of trigger criteria based on OAR dosimetry deformed form original treatment plan was established to assess replanning necessity. The feasibility and accuracy of automatic segmentation and dose prediction on CBCT were verified with rescan CT (rCT) and CBCT at the same time point.

**Results:**

The average dices of the automatic segmentation model for the lung, heart, and spinal cord were 0.92, 0.91, and 0.80, respectively. The predicted dose distributions on CBCT were close to mapped dose distributions. The ART trigger decision agreement between rCT and CBCT was 81.8%. CBCT with automatic segmentation OARs achieved an area under curve, accuracy, sensitivity, and specificity of 0.86, 0.82, 1.0, and 0.71 in the triggering of ART for EC patients, respectively.

**Conclusion:**

An automatic ART triggering procedure was established based on CBCT directly for EC patients underwent VMAT. It is a feasible and promising ART methods to improve the management of EC patients without additional patients appoints and resources.

## 1 INTRODUCTION

Esophageal cancer (EC) is a common malignant tumor in the digestive tract originated from the esophageal epithelium with a high fatality rate of more than 445 000 deaths per year.^1^ Concurrent chemoradiotherapy is the standard treatment for patients with inoperable EC,^2^, ^3^ in which radiotherapy plays an indispensable role with the aim to deliver a high therapeutic dose to the tumor while minimizing exposure to the surrounding healthy tissues.^4^ However, studies demonstrated that anatomy changes and/or target volume regressions of up to 30% were observed during the long‐term radiotherapy, which had a high potential to increase doses to surrounding organs at risk (OARs).^5^ Adaptive radiotherapy (ART) was introduced to incorporate patient‐specific anatomic changes into treatment plan modification to minimize overdose to surrounding healthy tissues while maintaining dose coverage in the target area.^6^, ^7^

Evaluation of the inter‐ and intra‐fractional tumor changes with various image modalities, such as rescan computed tomography (rCT), magnetic resonance imaging (MRI), cone‐beam CT (CBCT) and 4DCT, etc., is a common practice for ART.^8^, ^9^ However, ART involves repeated imaging, structure delineation, plan optimization and dose calculation that introduces additional demands on clinical resources and personal,^10^ especially rCT, MRI and 4DCT requires additional patient appointments, extra resource and staff time. The clinical adoption of linacs equipped with in‐room MRI scanners is still limited.^11^ Therefore, identifying appropriate triggers with in‐room verification imaging, CBCT, is critical to maximize the benefits of ART and prevent additional imaging dose to a certain extent for patients without compromising clinical resources or operations.^12^

On the other hand, direct use of CBCT for ART is limited due to the degraded image quality resulted from a series of scattering and noise artifacts,^13^ which renders direct target and OARs segmentation and dose calculation infeasible on CBCT.^14^ Deformable registration of pretreatment CT segmentations followed by manual human refinement was normally applied in the clinic on CBCT.^15^ Studies demonstrated that deep learning (DL) models are able to predict tumor changes based on daily CBCT images objectively so as to eliminate the propagation errors, deformation limitation and inter‐observer variations of human judgment.^16^ Studies further demonstrated that evaluation on the dosimetry changes resulted from anatomic changes is more clinically valuable during ART.^17^

The purpose of this study is to develop an automatic ART procedure by predicting and evaluating the patient's anatomical changes on OARs using DL based on CBCT to efficiently determine the time of triggering an adaptive plan, thus effectively balance the benefit and frequency of ART. A generative adversarial network (GAN) was first trained to segment automatically the OARs on CBCT‐based on CT‐CBCT registration; a dose prediction model was then trained and evaluated to predict the dose distribution of replanning plans on new OARs on CBCT. Finally, the ART replanning was triggered automatically based on the dose differences on OARs.

## 2 METHODS

### 2.1 Study design

**FIGURE 1:**
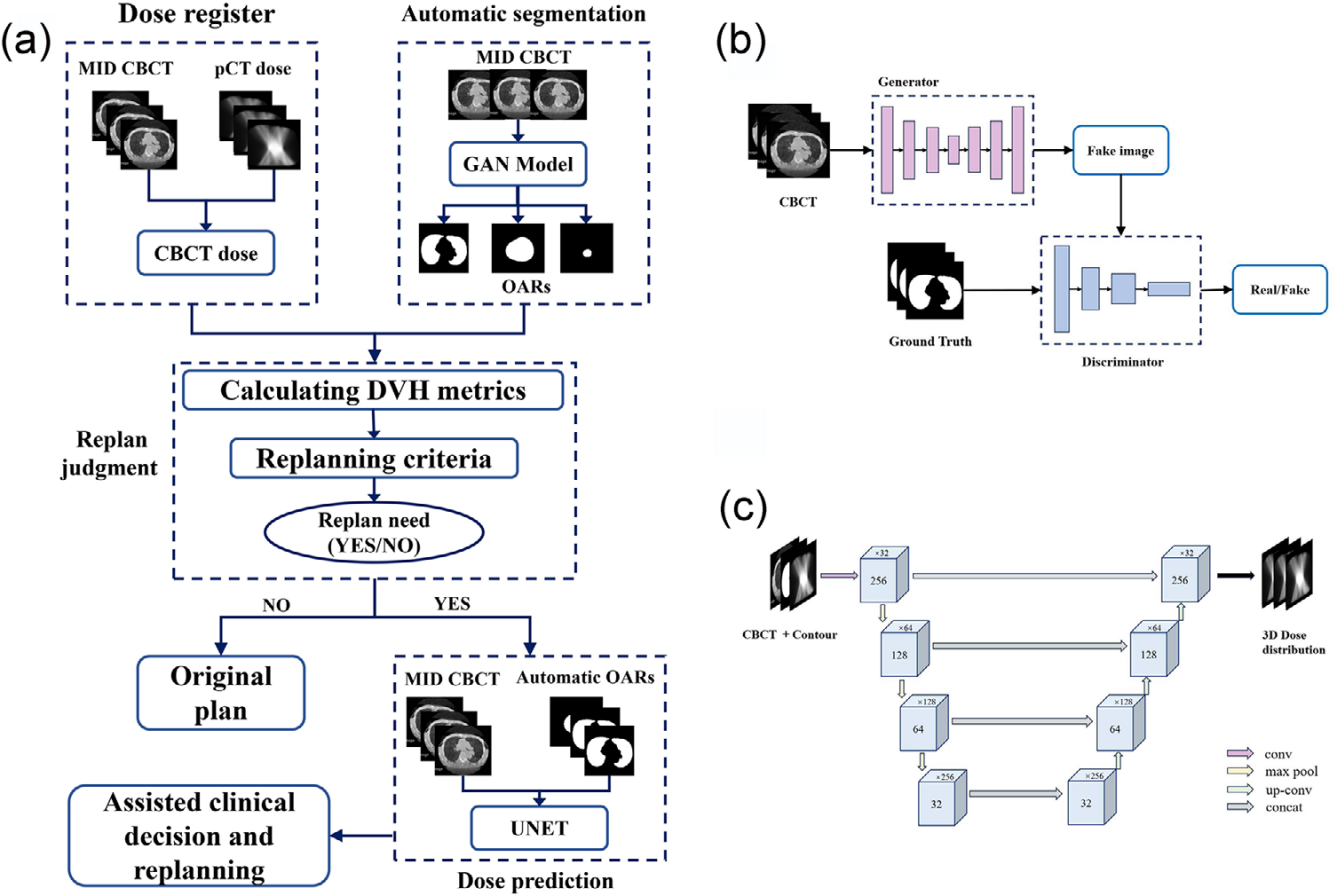
(a) Architecture of the automatic adaptive radiotherapy procedure and structures of the network used in this analysis; (b) the structure of generative adversarial network for automatic segmentation; (c) the structure of 3D Unet for dose prediction.

The framework to automatically determine the trigger of ART replanning based on current CBCT for EC patients during radiotherapy is shown in Figure 1a. A DL network was first trained to automatically segment OARs on the CBCT; the dose distribution registered from planning computed tomography (pCT) to CBCT to determine whether replanning was necessary based on established dose trigger criteria (see Section 2.5). The dose differences between plans of pCT and CBCT were calculated for each OAR separately to determine whether replanning was necessary based on established dose trigger criteria; the feasibility and accuracy of this procedure was verified with rCT and CBCT at the same time point.

### 2.2 Patients and images

Patients who underwent RT with CBCT in authors’ hospital from October 2021 to July 2023 were retrospectively reviewed. The automatic segmentation model and dose prediction model were trained on a dataset of EC patients treated by volumetric modulated arc therapy (VMAT) with contoured gross tumor volume (GTV), planned target volume (PTV), and OARs on pCT. VMAT plans were optimized in the Monaco Treatment Planning System (TPS) (Elekta, Crawley, UK) and Eclipse TPS (Varian Medical Systems, Palo Alto). Structures and doses on CBCT were propagated from pCT using MIM software (MIM Software Inc., Cleveland, OH, USA) with rigid registration and manual correction.^18^ Due to the limited scan coverage of CBCT, some images exhibited incomplete OAR contours. For these cases, during the data preprocessing stage, the OAR contours from the pCT were registered to the CBCT images to complete the OAR structures. An additional 11 patients with rCT images at the 20th fraction and corresponding mid‐treatment CBCT images were enrolled to validate this framework and ART trigger criteria. The radiation delivery systems employed in the present study comprised the Infinity radiotherapy platform (Elekta, Crawley, UK) and Edge radiosurgery system (Varian Medical Systems, Palo Alto). The CT images were acquired by a Philips CT scanner (Philips Healthcare, Best, Netherlands) with a slice thickness of 3 mm and a matrix of 512 × 512. The CBCT images were acquired by Varian On‐Board Imager and Elekta Infinity with 512 × 512 × 88 or 512 × 512 × 93 dimensions. This study was conducted in accordance with the Declaration of Helsinki and approved by the Research Ethics Committee (ECCR no. 2019059) of the author's hospital.

### 2.3 OARs automatic segmentation on CBCT

Patients were randomly divided into training and testing at a ratio of 7:3. CBCT images with propagated masks after registration were uniformly resampled to 256*256 for input. A GAN network consisting of a generator and a discriminator was trained and tested to automatically segment the OARs of EC patients on CBCT directly,^19^ as shown in Figure 1b. The CBCT images were input to the generator for segmentation, and the generated results were input to the discriminator along with the ground truth of CBCT with propagated segmentations from CT images. CT images contains pCT and rCT, and the scan times of CBCT for training were close to those of CT. Then the discriminant results were fed back to the generator for evaluation and improvement.

The automatically segmented ground truth is determined by a radiologist who modifies OARs deformed from treatment plan to CBCT. The stochastic gradient descent method was used to optimize the network with an initial learning rate for generator and discriminator set of 0.0002 and 0.00002, respectively. The cross‐entropy loss function and the Adam optimization algorithm were adopted and applied to minimize the loss between the generated contours and ground truth with total epochs of 300.^20^ The overall segmentation performance was evaluated by the pixel‐based dice similarity coefficient (DSC), Jaccard coefficient (JC), and average symmetric surface distance (ASSD).

### 2.4 Dose prediction on automatically segmented CBCT

A 3D Unet was applied for dose prediction on the CBCT images with target volumes and generated new OARs after GAN, as shown in Figure 1c the structure of this network.^21^ The training dataset for CBCT‐based dose prediction models included the first‐fraction CBCT scans, auto segmented OARs on CBCT, and GTV deformed from treatment plan as multi‐modal inputs. The dose distribution of treatment plan deformed to first‐fraction CBCT was trained as ground truth. The dosimetric prediction fidelity of the developed framework was systematically evaluated through comparative analysis of dose‐volume histogram (DVH) deviations. The absolute DVH discrepancy metric was mathematically expressed as:| δDVH| = | DVHp‐DVHpre |. DVHp denotes the treatment plan deformed to CBCT, and Dpre represents the model‐generated dose prediction.

Adam optimization algorithm was adopted to minimize the loss function value between the predicted and the mapped dose distribution with a batch size of 1 and a total training epoch of 300. The stochastic gradient descent method was used to optimize the network with an initial learning rate of 0.0002. Since sigmoid was used as the activation function, both CBCT images and dose profiles were normalized to 0–1 during training for better network convergence. The loss function of the dose prediction network combines the moment loss function in addition to the classical mean square error (MSE), moment loss was determined by the MSE differences between the predicted moments and ground truth.

### 2.5 Validation with rescan CT

Mid‐treatment CBCT images, rCT images of the same time point, and retreatment planning on rCT of 11 patients were collected to validate the accuracy of automatic contours, dose prediction, and the resulted ART trigger decision. Target volumes and OARs were delineated again on the rCT images by an experienced radiologist for replanning new radiotherapy treatment plans. Notably, truncated CBCT images were present in some cases. To address this, we employed rigid registration to propagate and map the complete external body contours and OAR (organs at risk) contours from the pCT onto the CBCT space, thereby filling the anatomical regions truncated in the CBCT and ensuring comprehensive dose calculation coverage. The criteria of ART trigger were determined by rCT following two rules: (1) the change of target volume is more than 15%; (2) the dose of OARs increased compared with those in pCT.

The ART trigger criteria using CBCT were determined by the dose differences of three OARs, lung, heart, and spinal cord, according to the recommendations of QUANTEC: (1) lung V5 < 60%, V20 < 30%, V30 < 20% (Vx, percentage of volume irradiated by X Gy); (2) for the heart: V30 < 40%, V40 < 30%, mean dose (D_mean_) < 26 Gy; (3) for the spinal cord: maximum dose (D_max_) < 45 Gy. If any of these criteria were violated, the replanning was considered for the patient.^22^

### 2.6 Statistical analysis

All the statistical analyses were performed using IBM SPSS Statistics software (version 19.0, IBM Inc., Armonk, NY, USA) and Python software (version 3.8, Anaconda Inc.). The DSC, JC, and ASSD were expressed as the mean ± standard deviation. The accuracy of dose prediction network under two OAR inputs was analyzed by paired *t*‐test, *P* < 0.05 indicates the difference is statistically significant.

## 3 RESULTS

A total of 136 EC patients treated by VMAT from October 2021 to July 2023 in authors’ hospital were enrolled in this study with a mean age of 68 years old. Two CBCT systems were applied with 108 and 28 patients acquired using Varian and Elekta, respectively, and a total of 12108 CBCT images were finally included in this study. The detailed characteristics of enrolled patients are summarized in Table S1.

**FIGURE 2:**
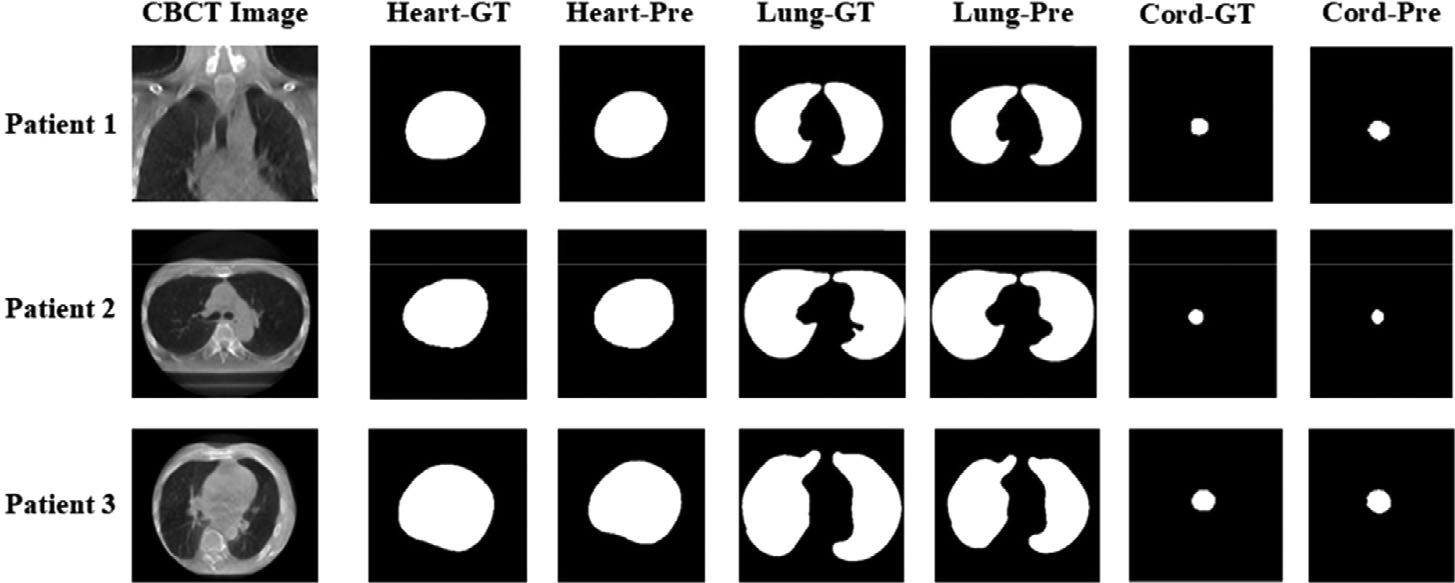
Segmentation results of GAN.

Figure 2 shows typical segmentation results with GAN for heart, lung, and cord. Visually, good agreement between segmented OARs on CBCT and ground truth contours on CT images was observed. The DSC for lung, heart, and cord was 0.80, 0.90, and 0.93, respectively. Detailed evaluation of automatic segmentation network for these three OARs are presented in Table 1.

**TABLE 1:** Mean DSC, JC, and ASSD, and their standard deviations for three models.

3D Model	DSC	JC	ASSD (mm)
Lung	0.927 ± 0.023	0.864 ± 0.040	0.954 ± 0.524
Heart	0.904 ± 0.028	0.826 ± 0.047	1.641 ± 0.642
Cord	0.800 ± 0.053	0.670 ± 0.072	1.195 ± 0.348

**FIGURE 3:**
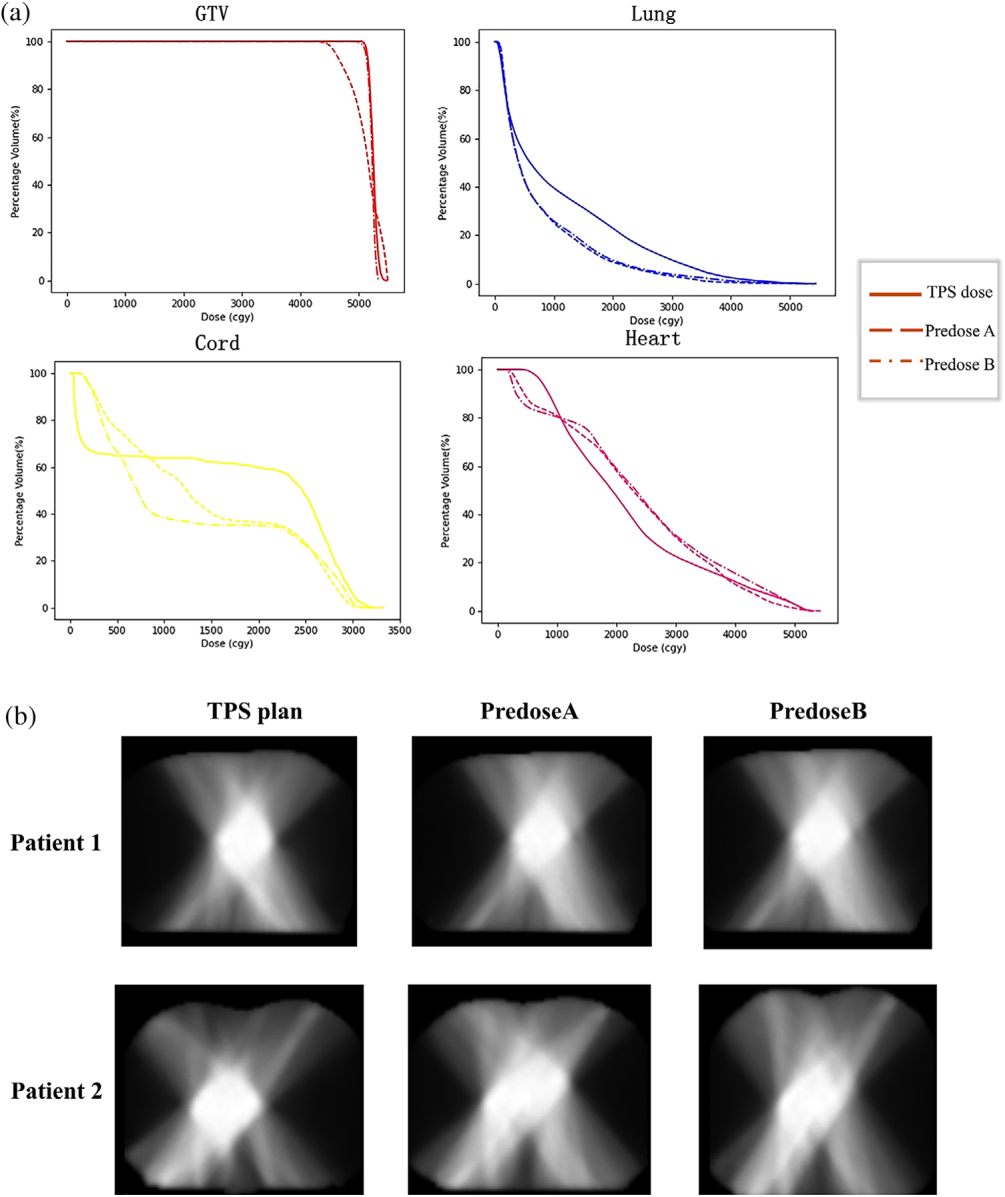
(a, b) DVH and dose distribution comparison for target volumes and OARs from plans from pCT (TPS dose) and predicted plans on CBCT with registration (Predose A) and automatically segmenting OARs (Predose B).

A typical DVH and dose distribution comparison between CBCT with automatically segmented OARs and with registered contours is shown in Figure 3a,b, where TPS dose represents the dose distribution from pCT registration to CBCT, and Predose B and Predose A represent the dose prediction results of OAR using registration and automatically segmenting OARs. The DVH of GTV in registered CBCT was close to that in TPS. The DVHs of lung, heart, and cord were close with no statistically significant differences between CBCT with registered and automatically contoured OARs. The detailed dose differences are presented in Tables 2 and 3. The predictive accuracy of the proposed dose distribution model is quantitatively assessed through DVH discrepancy analysis with the detailed δDVH discrepancies shown in Table 3.

**TABLE 2:** DVH comparison between CBCT with automatically segmented OARs and with registered contours.

		| δDVH | (mean ± std)		
Structure	Clinical indices	Mapped OAR	Auto‐segmented OAR	*P*‐value
GTV	D_2_(Gy)	1.344 ± 0.663	0.738 ± 0.489	1.01
	D_50_(Gy)	1.134 ± 0.544	0.592 ± 0.483	1.02
	V_95_(%)	0.061 ± 0.069	0.021 ± 0.044	0.92
	V_100_(%)	0.202 ± 0.149	0.045 ± 0.081	1.08
Lung	V_5_(Gy)	0.082 ± 0.074	0.092 ± 0.080	0.42
	V_20_(Gy)	0.081 ± 0.045	0.079 ± 0.047	0.45
				0.41
	V_30_(Gy)	0.046 ± 0.036	0.043 ± 0.036	
Heart	V_30_(Gy)	0.110 ± 0.116	0.114 ± 0.121	0.41
	V_40_(Gy)	0.060 ± 0.061	0.054 ± 0.057	0.11
Cord	D_max_(Gy)	3.803 ± 4.377	3.475 ± 4.522	0.39

**TABLE 3:** Comparison of DVH parameters of predicted dose and TPS dose in the validation set.

| δDose |										
	GTV			Lung			Heart			Cord
Patients	δD_2_ (Gy)	δD_50_ (Gy)	δV_95_ (%)	δV_100_ (%)	δV_5_ (%)	δV_20_ (%)	δV_30_ (%)	δV_30_ (%)	δV_40_ (%)	δD_max_
1	2.16	2.69	0.96	0	0.29	0.06	0.01	0.21	0.03	14.82
2	0.14	0.02	0	0.01	0.21	0.08	0.05	0.39	0.15	15.06
3	0.28	0.07	0.04	0	0.21	0.08	0.03	0.14	0.06	22.57
4	0.37	0.65	0	0	0.11	0.06	0.04	0.01	0.02	2.4
5	0.48	0.58	0	0	0.04	0.19	0.12	0.35	0.16	3.7
6	0.97	0.98	0	0	0.31	0.05	0.01	0.13	0.03	10.7
7	0.02	0.08	0	0	0.01	0.08	0.03	0.04	0.06	0.95
8	0.6	0.34	0.11	0.02	0.07	0.15	0.15	0.21	0.11	2.21
9	2.98	2.74	0	0.28	0.32	0.2	0.05	0.35	0.13	26.19
10	0.35	0.23	0	0	0.13	0.09	0.03	0.08	0.05	1.25
11	3.68	8.17	0.12	0	0.14	0.07	0.04	0.01	0.02	4.96
Mean	1.09	1.5	0.11	0.03	0.17	0.1	0.05	0.18	0.08	9.53
95% CI	0.74	1.43	0.17	0.05	0.06	0.03	0.03	0.08	0.03	5.32

The automatic ART trigger procedure was further validated with 11 validation cohorts with both CBCT and rCT images at the same time point. As shown in Tables 4 and 5, the average target volume was 44.16 ± 20.42 cm^3^ (range from 8.84 to 118.42 cm^3^) and 38.05 ± 17.36 cm^3^ (range from 8.70 to 101.84 cm^3^) in the pCT and rCT images, respectively. The rCT demonstrated that four patients showed a target volume reduction > 15%, and the replanning dose distribution is more suitable for patients in their current state that the dose on OARs decreased compared to rCT. While dose‐volume metrics evaluation based on CBCT with automatic segmentation showed that six patients demonstrated a deviation on lung V5, in which three patients had additional violation on lung V20, two patients had additional violation on cord dose, one patient deviated in heart V30. The agreement between rCT and CBCT was 81.8% in the judgment of triggering ART. As shown in Figure S1, further ROC analysis showed that CBCT with automatic segmentation OARs achieved an AUC of 0.86, accuracy (ACC) of 0.82, Sensitivity (SEN) of 1, and Specificity (SPE) of 0.71 in the triggering of ART for EC patients.

**TABLE 4:** Tumor changes assessed with planning and rescan CT images in the validation data set.

Patient	pCT tumor size(cm^3^)	Rescan fraction	rCT tumor size(cm^3^)	Volume changes (%)	Lung δV5 (%)	Lung δV20 (%)	Lung δV30 (%)	Heart δV30 (%)	Heart δV40 (%)	Cord δD_max_ (Gy)	ART trigger
1	26.05	25	25.97	−0.32	0.18	0.07	0.01	−0.01	−0.01	−4.04	0
2	8.84	20	8.7	−1.65	0.34	0.06	0.02	0	0	−7.36	0
3	29.09	20	28.66	−1.49	0.08	0.01	−0.01	−0.04	−0.03	−4.62	0
4	48.05	22	38.15	−20.6	−0.08	−0.04	−0.01	−0.13	−0.05	−2.46	1
5	28.24	21	18.01	−36.22	−0.03	−0.03	−0.01	−0.14	−0.06	−1.35	1
6	52.84	20	50.04	−5.29	0.01	−0.01	−0.01	0	0	−3.38	0
7	118.42	20	101.84	−14	−0.04	−0.02	−0.02	−0.04	−0.03	−0.77	0
8	31.29	22	26.93	−13.95	−0.03	−0.04	0	0.06	0.01	−1.16	0
9	46.58	25	45.29	−2.78	0.38	0.06	0.03	0	0	−3.75	0
10	22.02	21	17.63	−19.95	−0.12	0.01	0.01	−0.01	0.01	−0.59	1
11	74.36	20	57.35	−22.87	−0.02	−0.07	−0.03	−0.03	−0.05	−3.58	1

*Note*: a. δV = V_rCT_ ‐V_pCT,_ V_rCT_ represents the dose received by the volume on rCT, V_pCT_ is on pCT.

**TABLE 5:** Dose‐volume metrics evaluation for the trigger of ART with predicted results on CBCT with validation set.

	Heart		Lung					
Patient	V30 (%)	V40 (%)	V5 (%)	V20 (%)	V30 (%)	Cord D_max_ (Gy)	ART trigger rCT	ART trigger CBCT
1	1.35	0.69	38.18	13.87	3.71	28.51	0	0
2	0	0	38.63	17.52	8.4	33.08	0	0
3	15.02	7.07	49.39	17.83	6.39	29.8	0	0
4	18	0	**72.53**	10.16	2.75	**53.07**	1	1
5	**62.51**	**30.91**	**72.66**	**35.29**	17.34	39.34	1	1
6	0	0	6.35	0	0	12.13	0	0
7	23.93	0	**76.56**	18.19	0.91	25.45	0	1
8	34.56	21.05	**65.33**	27.6	13.25	**53.07**	0	1
9	0	0	25.36	8.38	2.6	41.66	0	0
10	39.48	19.19	**70.77**	**32.67**	10.03	39.99	1	1
11	34.53	22.01	**65.29**	**39.61**	5.94	39.99	1	1

*Note*: a: 1 indicates a need of trigger ART, 0 indicates no need of trigger ART, trigger replanning standard are shown in bold.

## 4 DISCUSSION

In this study, an automatic ART trigger decision procedure was proposed and validated for EC patients underwent RT by automatic OARs segmentation and dose prediction based on CBCT images using GAN and Unet, respectively. An AUC of 0.86 and ACC of 0.82 were achieved with this automatic ART trigger decision in comparison with the current clinical practice with rCT images.

Modern RT techniques, such as intensity modulated radiotherapy (IMRT) and VMAT, increased the precise of design and accurate irradiation to the target volume of EC patients, which in turn effectively reduces the dose to the surrounding normal tissues.^23^ However, tumor shrinkage caused positioning errors could result in a large variation in radiation doses and biological effects from the original planned treatment.^24^ In this study, the rCT for the 11 validation cohorts demonstrated an average tumor volume reduction of 15.21% (ranged from 0.32% to 43.35%) after 20th fraction for these EC patients underwent VMAT. These target volume reductions justified the application of ART for EC patients, as reported by Han et al.^25^ that at least 0.8 Gy and 1.2 Gy overdose per fraction resulted in lung and heart, respectively, for EC patients underwent external radiotherapy.

As the most widely used image modality in image guided radiotherapy (IGRT), CBCT is an idea image modality for ART to monitor target volume changes and to prevent OARs from receiving overdose and additional imaging dose to a certain extent. In this study, a GAN network was adapted to segment lungs, heart, and spinal cord automatically on CBCT relied on the registration between CBCT and pCT, and achieved an average DSC of 0.93, 0.90, and 0.80, respectively. This is very close to reported DSC of 0.96, 0.88, and 0.83 for lung, heart, and cord in the study of Dahiya et al., in which a conditional generative adversarial network (cGAN) was applied to translate CBCT to planning CT for OAR segmentation.^26^ The DSC for spinal cord was slightly lower than those of lungs and heart. A similar DSC of 0.84 ± 0.07 for the spinal cord was reported in the study of Dai et al., in which a fully automatic multi‐organ segmentation approach was investigated on CBCT with the aid of synthetic MRI.^27^

For the application of ART, the consequences of dosimetry differences resulted from anatomic changes are critical for the trigger of ART. In this study, the dose distributions on the CBCT with automatically contoured OARs and mapped OARs were predicted directly with 3DUnet. As shown in Figure 3 and Table 2, DVH and dose distributions of CBCT with automatically segmented OARs were very close to those calculated with registered contours. The GTV doses were close in CBCT in comparison with pCT in 11 validation cohorts, the differences were not statistical significance. The doses on lung, heart, and cord were very close between rCT and CBCT with automatic segmentations. Similarly, Tsironi et al. demonstrated a real time dose difference of 2.5% ± 1.4% and 2.4 % ± 2.1% in the heart and lungs between predicted dose using chest CBCT and Monte Carlo simulation, respectively.^28^ These studies indicated that feasibility and accuracy of CBCT‐based OAR segmentation and dose prediction for ART.

Direct prediction and evaluation on the dose‐volume metrics differences resulted from anatomic changes is preferred for triggering of ART. Nyeng et al. used rCT at middle fraction to evaluate the changes of target volume and their dose consequences to trigger ART according to the decrease of V95% of CTV > 1% or PTV > 3%, CBCT was used to conform these discrepancies only.^29^ In this study, CBCT was applied directly for automatic segmentation and dose‐volume metrics evaluation for the triggering of ART. According to the validation with rCT at the same time point, an AUC of 0.86, accuracy (ACC) of 0.82, Sensitivity (SEN) of 1, and Specificity (SPE) of 0.71 was achieved with our proposed automatic ART triggering procedure with CBCT.

The present study has several limitations. First, the validation cohort was limited to 11 cases with both rCT and CBCT available, which constrained our ability to comprehensively verify the feasibility and accuracy of the proposed automatic ART triggering procedure for OARs. Future validation of our method will require more clinical data containing both rCT and CBCT from multiple centers, and should also incorporate the GTV into the analysis. Second, the use of deformed dose distributions as ground truth represents a simplification of the actual physical dose delivery process. Although rigid registration was employed to minimize geometric uncertainties, this approach does not fully account for scatter conditions and beam path heterogeneities inherent to CBCT imaging geometry. Third, the prediction workflow was designed to flag patients for physician review rather than to automatically trigger replanning orders; consequently, dose thresholds (e.g., lung V_5) can be adjusted according to institutional protocols. Specifically, higher thresholds are recommended for offline ART to avoid unnecessary workload, whereas lower thresholds may be acceptable for online adaptive platforms—a direction we have identified for future development. Forth, spinal cord DSC of 0.80 introduces delineation uncertainty, and combined with registration errors in high‐dose‐gradient regions, may not be suitable for replanning judgment factor. Fifth, the automatic segmentation and dose prediction models were relied on the registration between planning CT and CBCT, which may introduce unavoidable registration errors into the procedure. Generating synthetic CT images from CBCT using DL algorithms to improve the image quality of CBCT may be tried in our future study to improve the image quality of CBCT for automatic segmentation and direct dose calculation. Finally, the strategy of compensating for CBCT truncation by mapping body contours from the planning CT fails to reflect true anatomical changes, which may compromise the accuracy of our dose assessment and replanning decisions.

In this study, an automatic ART triggering and application procedure was applied based on CBCT images direct for EC patients underwent VMAT. It is a feasible and promising ART methods to improve the management of EC patients without additional patients appoints and resources.

## AUTHOR CONTRIBUTIONS

Juebin Jin, Jiayi Xu, Xiance Jin, and Yongqiang Zhou designed, supervised the project. Yapeng Liu, Kai Shao, Yangyang Zhang, Juebin Jin, and Jiayi Xu analyzed the data. Juebin Jin and Jiayi Xu performed and analyzed most of statistical experiments. Jiayi Xu, Yongqiang Zhou, Huawei Yan, Zhiwei Wang, and Li Lin acquired data. Yongqiang Zhou and Xiance Jin verified the accuracy of the data analysis. All authors have read and approved the final manuscript.

## CONFLICT OF INTEREST STATEMENT

The authors declare no conflicts of interest.

## Supporting information



Supporting Information

## Data Availability

The datasets used and/or analyzed during the current study are available from the corresponding author on reasonable request.
