# Supplementary material for: Automatic adaptive radiotherapy triggering based on CBCT using deep learning for esophageal cancer underwent volumetric modulated arc therapy
Source: J Appl Clin Med Phys. 2026 Jun 10;27(6):e70651. doi: 10.1002/acm2.70651 (PMC13250693; doi:10.1002/acm2.70651)
Supplement: Supplementary file 1 — Supporting Information [file ACM2-27-e70651-s001.docx]

**1. Patients and images**

Patients underwent RT with CBCT in authors’ hospital from October 2021 to July 2023 were retrospectively reviewed. The automatic segmentation model and dose prediction model were trained on a dataset of EC patients treated by volumetric modulated arc therapy (VMAT) with contoured gross tumor volume (GTV), planned target volume (PTV), and OARs on pCT. A total of 136 EC patients were randomly split into training and testing sets at a 7:3 ratio. VMAT plans were optimized in the Monaco Treatment Planning System (TPS) (Elekta, Crawley, U.K.) and Eclipse TPS (Varian Medical Systems, Palo Alto),“Chinese Guidelines for Diagnosis and Treatment of Esophageal Cancer” (2022 Edition) used to evaluate plan quality used for training. Structures and doses on CBCT were propagated from pCT using MIM software (MIM Software Inc., Cleveland, OH, USA) with rigid registration and manual correction. Additional 11 patients with rCT images at the 20th fraction and corresponding mid-treatment CBCT images were enrolled to validate this framework and ART trigger criteria. The radiation delivery systems employed in the present study comprised the Infinity™ radiotherapy platform (Elekta, Crawley, U.K.) and Edge™ radiosurgery system (Varian Medical Systems, Palo Alto). The CT images were acquired by a Philips CT scanner (Philips Healthcare, Best, Netherlands) with a slice thickness of 3 mm and a matrix of 512 × 512. A total of 12,108 CBCT images (512 × 512 × 88 or 512 × 512 × 93 dimensions) were acquired using two imaging systems: 108 patients were scanned with the Varian On-Board Imager and 28 with the Elekta Infinity system. CBCT scans were acquired using Varian (125 kV, 268 mAs) and Elekta (100 kV, 18.1 mAs) linear accelerators, respectively. The detailed characteristics of enrolled patients were summarized in Table.S1. This study was conducted in accordance with the declaration of Helsinki and approved by the Research Ethics Committee (ECCR no.2019059) of the author’s hospital.

Table.S1 Patient’s characteristics and patient numbers included

| Characteristics | Description | Entire cohort (n=136) |
| --- | --- | --- |
| Sex | Male  Female | 123 (90%)  13 (10%) |
|  |  |  |
|  | 40~59  60~80 | 21 (15%)  92 (68%) |
| Age at diagnosis (y) |  |  |
|  | 80+ | 23 (17%) |
| TPS system | Monaco  Varian | 108 (79%)  28 (21%) |
|  |  |  |
|  | Cervical  Thoracic  Abdominal | 20 (14%)  92 (68%)  24 (18%) |
| Tumor Location |  |  |
|  |  |  |
| Total dose | 36~50.4 Gy  54 Gy  60Gy | 70 (52%)  30 (22%)  36 (26%) |

**2. Results**

Figure.S1 shows the ROC curve of the CBCT auto-segmentation OAR in triggering ART in EC patients, with a final AUC of 0.86, accuracy (ACC) of 0.82, sensitivity (SEN) of 1, and specificity (SPE) of 0.71.


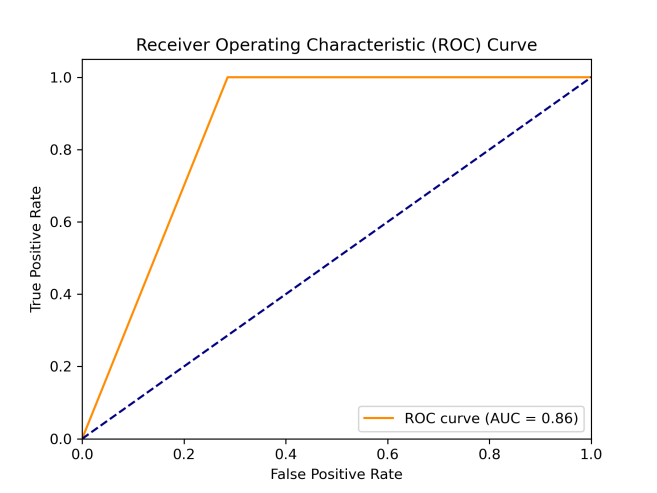


Figure.S1 The performance of automatic ART triggering procedure based on CBCT with receiver operation curves.
